# Comparison of extracorporeal shock wave therapy targeting the posterior myofascial chain and plantar tenderness points versus plantar tenderness points alone in patients with plantar fasciitis: a randomized controlled trial

**DOI:** 10.3389/fmed.2026.1943850

**Published:** 2026-09-11

**Authors:** Shichong Wang, Siyifan Chen, Han Yang, Baoshan Xu, Chuan Guo, Gaojian Tao, Ying Shen

**Affiliations:** 1 Gulou Clinical Medical College, Nanjing Medical University, Nanjing, China; 2 Department of Rehabilitation Medicine, The First Affiliated Hospital with Nanjing Medical University, Nanjing, China; 3 Department of Rehabilitation Medicine, The Affiliated Suqian Hospital of Xuzhou Medical University, Suqian, China; 4 Department of Pain Medicine, Nanjing Gulou Hospital, Nanjing, China

**Keywords:** clinical, extracorporeal shock wave therapy, myofascial chain, plantar fasciitis, trigger point

## Abstract

**Aim:**

Plantar fasciitis is a common cause of heel pain. Although extracorporeal shock wave therapy (ESWT) targeting plantar tenderness points is widely used, outcomes remain unsatisfactory in some patients. This study aimed to compare the clinical efficacy of ESWT targeting both posterior myofascial-chain trigger points and plantar tenderness points with that of conventional ESWT targeting plantar tenderness points alone in patients with plantar fasciitis.

**Methods:**

Eighty patients with plantar fasciitis were randomly assigned to a control or treatment group (*n* = 40 per group). The control group received conventional ESWT applied only to the local plantar tenderness point, whereas the treatment group received ESWT applied to both gastrocnemius trigger points and the plantar tenderness point. Treatment was administered once weekly for four consecutive weeks. Participants were evaluated at baseline and at 1 and 3 months after treatment. Outcomes included the American Orthopaedic Foot and Ankle Society (AOFAS) ankle–hindfoot score, visual analog scale (VAS), strain ratio (SR), plantar fascia thickness, and Y-Balance Test (YBT) score. Repeated-measures analysis of covariance was used to examine group, time, and group × time effects, with adjustment for prespecified covariates.

**Results:**

Significant group × time interactions were observed for VAS, AOFAS, and YBT scores. *Post hoc* comparisons showed statistically significantly lower VAS scores and higher AOFAS and YBT scores in the treatment group than in the control group at follow-up (all *P* < 0.01). These statistical differences should be interpreted separately from clinical relevance. For SR and plantar fascia thickness, the main effect of time was significant (both *P* < 0.001), whereas the group and group × time effects were not significant (all *P* > 0.05).

**Conclusion:**

The combined protocol was associated with greater short-term improvements in pain, ankle–hindfoot function, and dynamic balance than plantar-focused ESWT alone. The clinical importance of some between-group differences remains uncertain. These findings do not establish a myofascial-chain mechanism or treatment safety, and conclusions are restricted to short-term comparative effectiveness.

**Clinical Trial Registration:**

https://www.chictr.org.cn/index.html, identifier ChiCTR250010696

## Introduction

Plantar fasciitis is the most common cause of heel pain and is considered a chronic degenerative disorder of the plantar fascia, often accompanied by aseptic inflammation at its medial calcaneal attachment (1). Patients typically experience sharp, stabbing pain in their heels upon taking their first steps in the morning or after prolonged rest. Although the pain often subsides after brief activity, it tends to recur or worsen with prolonged standing or walking, substantially impairing daily function and mobility. The etiology of plantar fasciitis is multifactorial, involving chronic mechanical stress, abnormal foot biomechanics (e.g., flat or high arched foot), obesity, diabetes, and age-related degeneration (2–6). Conventional treatment strategies primarily target the plantar fascia insertion at the calcaneus, which is commonly regarded as the principal source of pain. Current conservative management options include rest, stretching exercises, kinesiology taping, orthotic insoles, local corticosteroid injections, and extracorporeal shock wave therapy (ESWT) (7–10). Among these options, ESWT has emerged as a first-line therapy for plantar fasciitis refractory to standard conservative treatment because it is noninvasive and has demonstrated clinical efficacy (11, 12). Its therapeutic effects are attributed to the mechanical stimulation of tissues, which promotes tissue regeneration, enhances inflammation resolution, releases adhesions, and inhibits nociceptive signaling (13–15).

Clinically, however, some patients treated with conventional ESWT restricted to local plantar tenderness points experience suboptimal outcomes or symptom recurrence (16). One reasonable interpretation for these phenomena is that plantar fasciitis may not be purely a local pathology, but could also be associated with upstream biomechanical dysfunction and myofascial chain imbalances that sustain the symptoms (17–19). In recent years, myofascial-chain and myofascial trigger-point concepts have been proposed as possible frameworks for understanding some chronic pain conditions. According to myofascial-chain theory, muscles and fascia may be linked through proposed biomechanical pathways. The “superficial back line” is described as a continuous pathway involving the plantar fascia, Achilles tendon, gastrocnemius, hamstrings, sacrotuberous ligament, erector spinae, and galea aponeuroticasacrotuberous ligament, and galea aponeurotica (20). Abnormal plantar-fascia tension has been hypothesized to propagate proximally along this pathway, while dysfunction in proximal structures, such as the triceps surae or hamstrings, may increase plantar-fascia loading (21–23).

Myofascial trigger points are hyperirritable nodules within taut bands of skeletal muscle that may produce referred pain and functional impairment (24). Latent or active trigger points may occur in the gastrocnemius and soleus muscles, with referred-pain patterns that can extend to the heel and potentially aggravate plantar-fasciitis symptoms (25). Failure to address proximal myofascial abnormalities could be one explanation for suboptimal outcomes after treatment directed only at plantar tenderness points. Evaluating and treating gastrocnemius trigger points may therefore offer a broader therapeutic strategy; however, the proposed transmission of pathological force along a myofascial chain remains a hypothesis. Previous studies have primarily examined ESWT for localized plantar pathology (26, 27) or manual myofascial-release techniques (28), whereas the systematic incorporation of myofascial-chain concepts into ESWT protocols targeting trigger points requires further investigation.

Accordingly, this randomized controlled trial was designed to compare the clinical effectiveness of ESWT applied to both gastrocnemius trigger points and plantar tenderness points with that of conventional plantar-focused ESWT in patients with plantar fasciitis. In addition to evaluating a combined ESWT protocol informed by myofascial-chain concepts, the study used a multidimensional framework encompassing structural measures (ultrasound-derived strain ratio and plantar fascia thickness), ankle–hindfoot function (AOFAS score), pain (VAS), and dynamic balance (YBT).

## Materials and methods

### Sample size calculation

Based on the between-group difference in post-treatment VAS pain scores, the sample size was estimated. We referred to the randomized controlled trial by Moghtaderi et al (29) on extracorporeal shock wave therapy in patients with plantar fasciitis, in which pain intensity was evaluated utilizing a 100-mm visual analogue scale (VAS). Grounded on the between-group difference in VAS pain scores at 8 weeks after treatment reported in that study, the standardized effect size was calculated to be approximately Cohen's *d* = 0.995.

Using this effect size, the sample size was calculated with G*Power version 3.1.9.7. The analysis was specified as a two-sided comparison of two independent means, with 90% power, *α* = 0.05, an effect size of *d* = 0.995, and a 1:1 allocation ratio. The calculation indicated that at least 23 participants were required per group (46 in total), corresponding to an actual power of 0.9098.

Allowing for 20% attrition due to loss to follow-up, withdrawal, or incomplete data, the required sample was increased to at least 29 participants per group (58 in total). To further improve statistical power and robustness, the target sample was set at 40 participants per group (80 in total). Ultimately, 37 participants in the control group and 38 in the treatment group were included in the final analysis (75 participants in total).

### Study participants

**Figure 1:**
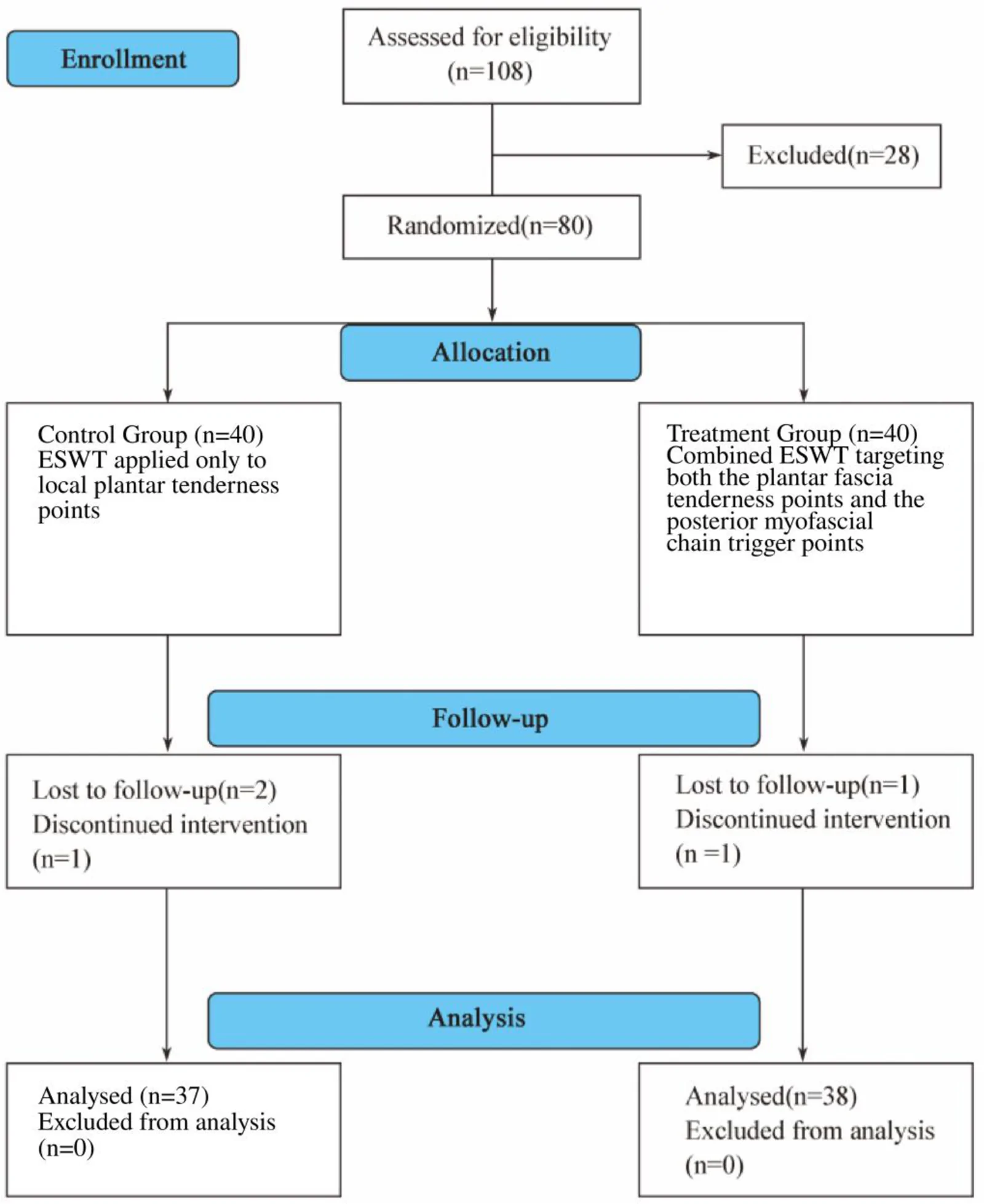
Flow diagram of participant selection, randomization, follow-up, and analysis.

Eighty patients with plantar fasciitis who were admitted to our hospital between August 2,025 and February 2026 were recruited. A computer-generated random sequence was used to assign 40 participants to each group. The control group received conventional ESWT at local plantar tenderness points, whereas the treatment group received ESWT at both gastrocnemius trigger points and plantar tenderness points. Figure 1 presents participant recruitment, allocation, follow-up, and analysis.

### Inclusion criteria

Participants were eligible if they met all of the following criteria: (1) age 18–70 years; (2) plantar fasciitis diagnosed according to established clinical criteria (30); (3) pain of any intensity at the proximal plantar fascia or calcaneal insertion; (4) unilateral symptoms; (5) no calcaneal spur on radiography; and (6) voluntary participation with written informed consent.

### Exclusion criteria

Participants were excluded if they met any of the following criteria: (1) heel pain attributable to lumbar spine disorders, peripheral neuropathy, or another ankle or foot disorder (e.g., fracture, arthritis, tendon rupture, or tarsal tunnel syndrome); (2) skin lesions, infection, or tumors of the foot; (3) previous surgery or severe deformity of the affected lower limb; (4) severe cardiovascular or cerebrovascular disease, a coagulation disorder, or active malignancy; (5) severe psychiatric or cognitive impairment preventing cooperation with treatment or assessment; (6) pregnancy or lactation; or (7) concurrent participation in another clinical trial that could affect study outcomes.

### Methods

This randomized controlled trial of a nonpharmacological shock-wave intervention was designed and reported in accordance with the CONSORT 2025 Statement.

### Treatment protocols

#### Control group

Radial ESWT was administered using a Swiss EMS shock-wave device (Swiss DolorClast Master). Participants received conventional ESWT at the local plantar-fascia tenderness point (2–3 bar, 10 Hz, 2,000 impulses). Sessions were performed once weekly for four consecutive weeks. After each session, participants were instructed to avoid strenuous physical activity, thermotherapy, and topical heating agents for 72 h. All participants were also instructed to perform home stretching and relaxation exercises. For plantar-fascia stretching, participants sat with the affected foot resting on the opposite knee, held the heel with one hand, and gently pulled the forefoot and toes with the other hand. For triceps-surae stretching, participants lay supine with a towel placed under the sole, held both ends of the towel, kept the knee extended, and raised the lower leg. Participants performed three sets daily, with three repetitions per set and each stretch held for 30 s; each set lasted approximately 3–5 min.

#### Treatment group

**Figure 2:**
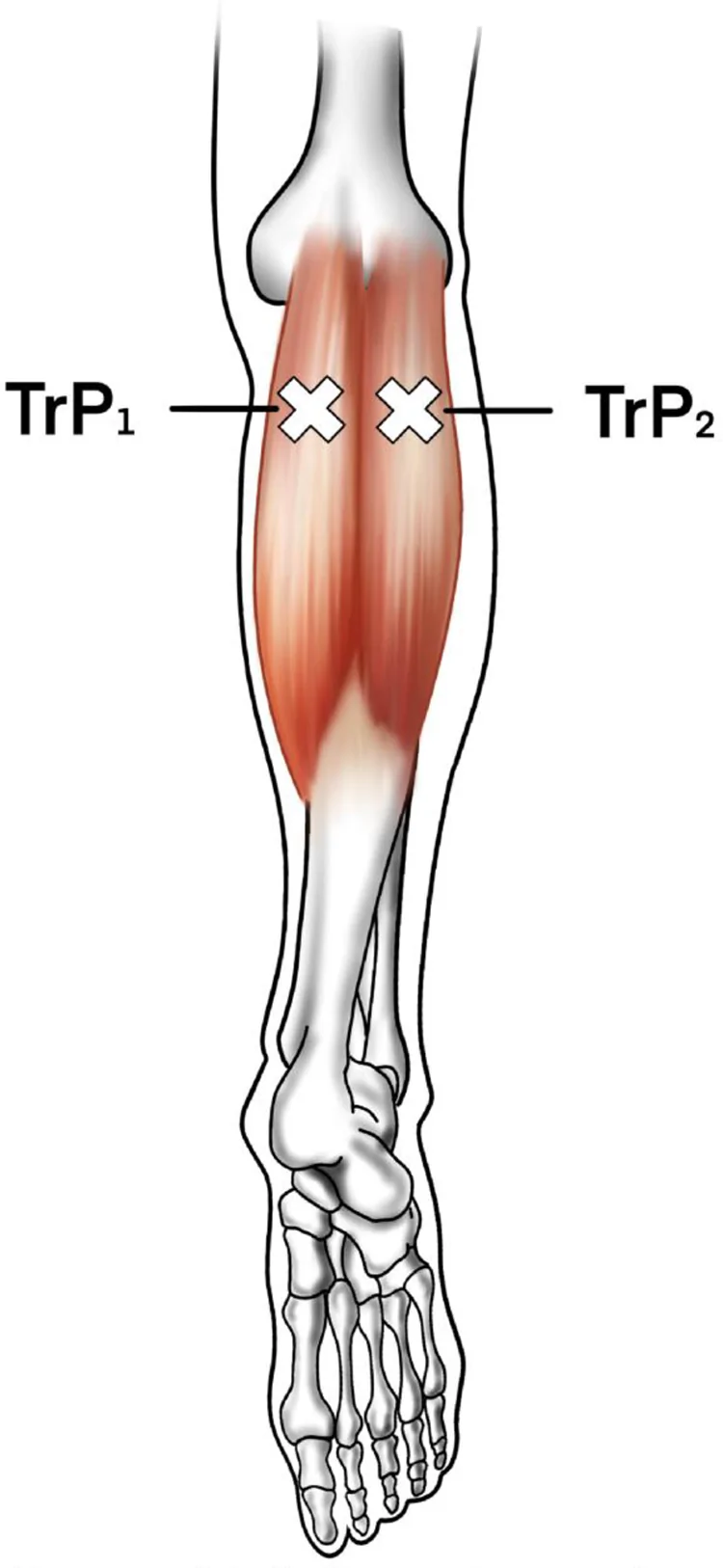
Trigger points in the gastrocnemius muscle targeted during extracorporeal shock wave therapy.

Participants in the treatment group received combined ESWT at the plantar-fascia tenderness point and gastrocnemius trigger points. Trigger points were identified with the participant prone, the knee fully extended, and the ankle in a neutral position. The medial and lateral gastrocnemius heads were systematically palpated for taut bands and the points of greatest tenderness. In this trial, one trigger point in each gastrocnemius head was treated (Figure 2). The treatment parameters were otherwise identical to those used in the control group, and 2,000 impulses were delivered per session: 500 to each gastrocnemius trigger point and 1,000 to the local plantar-fascia tenderness point. Post-treatment precautions and stretching exercises were identical in both groups. All participants included in the analysis completed the scheduled treatment sessions and were instructed not to receive other interventions for plantar fasciitis during the trial.

### Outcome measures

To comprehensively evaluate the therapeutic effects of the interventions, a multidimensional outcome assessment framework encompassing pain, function, structural characteristics, and dynamic balance was employed.

### Primary outcome

#### Pain intensity

Pain intensity was assessed using a visual analog scale (VAS) (31). Participants rated heel-pain intensity on a 10-cm horizontal line anchored by “0 = no pain” and “10 = worst pain imaginable,” with higher scores indicating greater pain intensity.

### Secondary outcomes

#### Ankle–hindfoot function

The American Orthopaedic Foot and Ankle Society (AOFAS) ankle–hindfoot scale was used to assess function (32). This 100-point scale assesses pain, function, and alignment, with higher scores indicating better ankle–hindfoot function. Scores are commonly categorized as poor (<50), fair (50–74), good (75–89), and excellent (90–100).

### Dynamic balance ability

**Figure 3:**
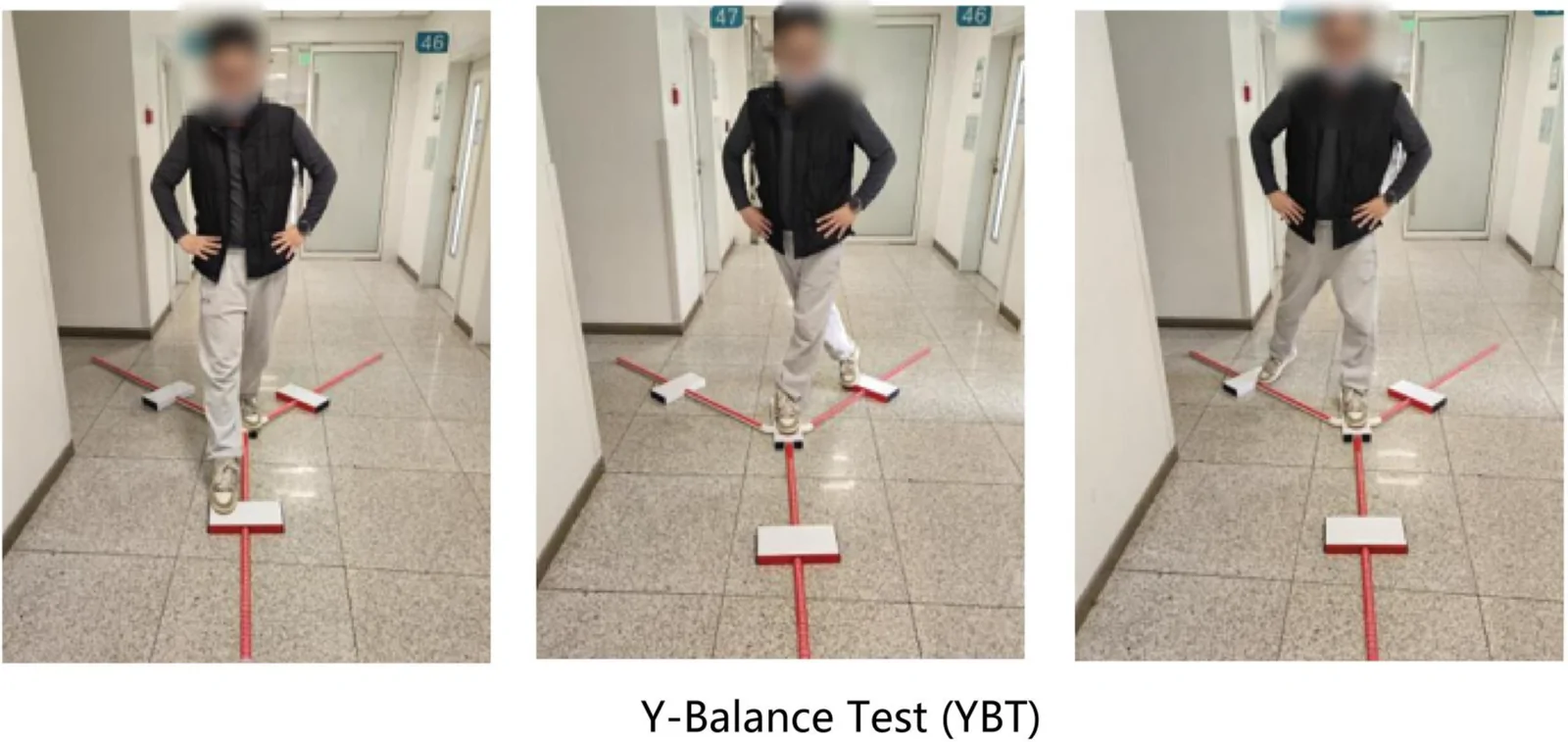
Performance of the Y-Balance Test.

The Y-Balance Test (YBT) assesses dynamic balance by measuring the maximum reach of the nonstance limb in three directions (anterior, posteromedial, and posterolateral) while the participant stands on one leg. A leg-length difference of ≥1 cm can affect the result (33). The YBT composite reach score was calculated as the sum of the three reach distances divided by three times the limb length, multiplied by 100% (Figure 3).

### Ultrasonographic parameters

**Figure 4:**
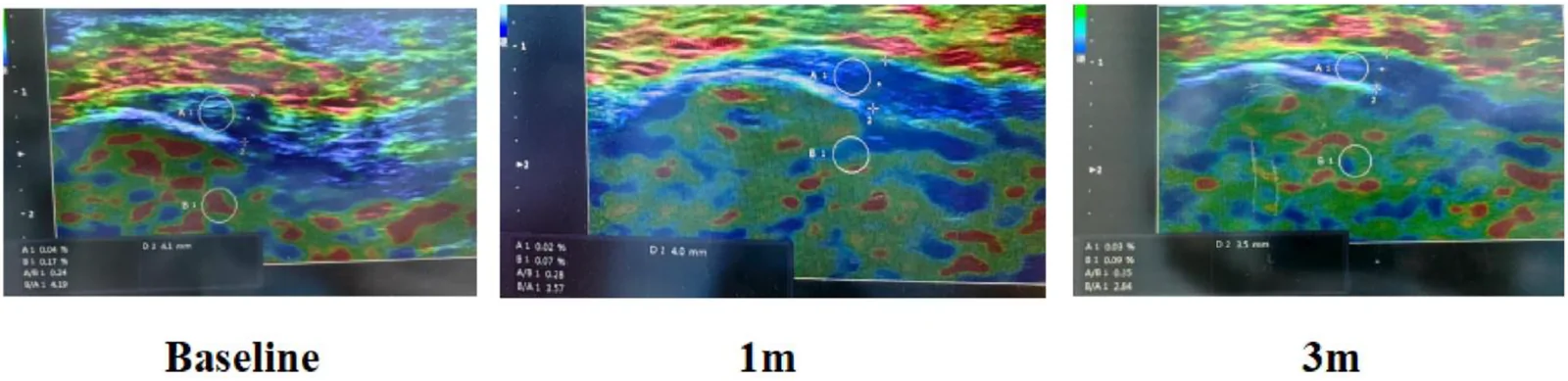
Representative ultrasonographic measurements of plantar fascia strain ratio and thickness.

Using a KONICA MINOLTA ultrasound diagnostic system, musculoskeletal two-dimensional and elastography modes were applied with a linear probe (8–15 MHz) to measure the strain ratio (SR) and plantar fascia thickness at the calcaneal-fascial junction while the patient was in a prone position (Figure 4). Probe-compression force was not quantitatively monitored or instrument-controlled during sonoelastography acquisition; therefore, no prespecified numerical force target was used.

### Blinding procedure

A single-blind design with allocation concealment was used.

#### Allocation concealment

An independent researcher generated the computerized randomization sequence. Group assignments were placed in opaque, sequentially numbered, sealed envelopes. The treating physiotherapist opened the corresponding envelope immediately before the first treatment session to determine group allocation.

#### Blinding of outcome assessors and statistician

Outcome assessors were blinded to group allocation. VAS, AOFAS, ultrasound, and YBT assessments at all time points were performed by evaluators who were not involved in treatment delivery and were unaware of group assignment. The statistician remained blinded to group allocation until the analysis was completed.

### General information

Baseline demographic and clinical characteristics, including age, sex, body mass index (BMI), disease duration, and affected side, were collected before intervention (Table 1).

**Table 1:** Baseline demographic and clinical characteristics of the two groups.

Variable	Control (*n* = 37)	Treatment (*n* = 38)	*P*
Age (years)	44.65 ± 11.53	44.66 ± 14.04	0.998
Height (cm)	167.86 ± 8.37	167.00 ± 8.62	0.661
Weight (kg)	67.24 ± 12.05	65.92 ± 11.89	0.634
BMI (kg/m^2^)	23.72 ± 2.91	23.49 ± 2.91	0.740
Duration (weeks)	37.51 ± 25.47	36.63 ± 19.40	0.867
Baseline VAS	5.16 ± 0.90	5.03 ± 0.72	0.472
Baseline AOFAS	58.65 ± 8.13	59.68 ± 8.91	0.600
Baseline strain ratio	0.48 ± 0.14	0.46 ± 0.16	0.675
Baseline thickness (mm)	3.91 ± 0.78	3.87 ± 0.69	0.804
Baseline YBT	0.72 ± 0.07	0.72 ± 0.09	0.741
Sex F/M	17/20	19/19	0.904
Affected side: L/R	17/20	19/19	0.904

Data are presented in the form of mean ± SD or number. BMI, body mass index; AOFAS, American Orthopaedic Foot and Ankle Society ankle–hindfoot score; VAS, visual analogue scale; SR, strain ratio; YBT, Y Balance Test; F/M, female/male; L/R, left/right.

### Statistical analysis

Statistical analyses were performed using SPSS version 25.0 (IBM Corp., Armonk, NY, USA). Normality of continuous variables was assessed using the Shapiro–Wilk test. Normally distributed variables are presented as means and standard deviations (SDs), and nonnormally distributed variables as medians and interquartile ranges. Categorical variables are reported as frequencies and percentages. Baseline characteristics were summarized descriptively. Between-group comparisons at baseline used independent-samples t tests or Mann–Whitney U tests for continuous variables, as appropriate, and *χ*^2^ or Fisher exact tests for categorical variables.

The effects of group, time, and the group × time interaction on each outcome were examined using repeated-measures analysis of covariance (RM-ANCOVA). The primary effect of interest was the group × time interaction, indicating whether changes over time differed between groups. Age, sex, BMI, disease duration, and affected side were included as prespecified covariates. Sphericity was assessed using Mauchly's test, and Greenhouse–Geisser correction was applied when the assumption was violated.

When a significant group × time interaction was detected, simple-effects analyses examined between-group differences at each time point and within-group changes over time. The Benjamini–Hochberg procedure was used to control for multiple comparisons. Adjusted between-group mean differences and 95% confidence intervals (CIs) were reported where appropriate. Effect sizes were expressed as Cohen's *d* for between-group differences at follow-up and partial eta squared (*η*p^2^) for main and interaction effects. Values of *η*p^2^ of 0.01, 0.06, and 0.14 were interpreted as small, medium, and large, respectively.

## Results

### Baseline characteristics

A total of 75 of the 80 randomized participants (93.8%) were included in the complete-case analysis: 37 in the control group and 38 in the treatment group. Three participants in the control group and two in the treatment group were not included because complete longitudinal outcome data were unavailable. Baseline demographic and clinical characteristics—including height, weight, age, BMI, disease duration, VAS pain score, SR, plantar fascia thickness, YBT score, AOFAS ankle–hindfoot score, sex, and affected side—were comparable between groups (all *P* > 0.05) (Table 1).

### Pain intensity (VAS scores)

Baseline VAS pain scores did not differ significantly between the two groups (*P* = 0.472). RM-ANCOVA showed significant main effects of group (*F* = 8.63, *P* = 0.003, *η*p^2^ = 0.038) and time (*F* = 362.29, *P* < 0.001, *η*p^2^ = 0.626), and a significant group × time interaction (*F* = 12.13, *P* = 0.002, *η*p^2^ = 0.053). Simple-effects analyses showed a statistically significant between-group difference in VAS pain at 1 month, with an adjusted mean difference of −0.63 points (95% CI: −1.03 to −0.23; BH-adjusted *P* = 0.006; Cohen's *d* = 0.72). At 3 months, the adjusted mean difference was −0.93 points (95% CI: −1.43 to −0.43; BH-adjusted *P* = 0.001; Cohen's *d* = 0.85) (Tables 2, 3). These statistical differences do not by themselves establish clinical superiority.

**Table 2:** Main model summary table.

Variable	Control			Treatment			Effect	*F*	df	*P*	*P*_FDR	*η*p^2^
	Pre	1 Month	3 Months	Pre	1 Month	3 Months						
VAS	5.16 ± 0.90	2.97 ± 0.87	2.16 ± 1.12	5.03 ± 0.72	2.34 ± 0.88	1.24 ± 1.05	Group	8.63	1, 216	0.003	0.005	0.038
							Time	362.29	2, 216	<0.001	<0.001	0.626
							Group × Time	12.13	2, 216	0.002	0.003	0.053
AOFAS	58.65 ± 8.13	73.43 ± 7.07	82.57 ± 6.10	59.68 ± 8.91	79.79 ± 6.24	90.05 ± 6.60	Group	14.78	1, 216	<0.001	<0.001	0.064
							Time	349.39	2, 216	<0.001	<0.001	0.618
							Group × Time	14.42	2, 216	<0.001	0.001	0.063
SR	0.48 ± 0.14	0.52 ± 0.14	0.67 ± 0.15	0.46 ± 0.16	0.58 ± 0.15	0.71 ± 0.15	Group	2.49	1, 216	0.114	0.132	0.011
							Time	66.79	2, 216	<0.001	<0.001	0.236
							Group × Time	4.57	2, 216	0.102	0.127	0.021
PFT (mm)	3.91 ± 0.78	3.64 ± 0.76	3.19 ± 0.78	3.87 ± 0.69	3.54 ± 0.60	3.01 ± 0.69	Group	0.20	1, 216	0.652	0.652	0.001
							Time	72.45	2, 216	<0.001	<0.001	0.251
							Group × Time	1.37	2, 216	0.505	0.541	0.006
YBT Score	0.72 ± 0.07	0.76 ± 0.06	0.80 ± 0.05	0.72 ± 0.09	0.83 ± 0.08	0.87 ± 0.06	Group	19.62	1, 216	<0.001	<0.001	0.083
							Time	100.07	2, 216	<0.001	<0.001	0.317
							Group × Time	40.18	2, 216	<0.001	<0.001	0.157

Data are presented as mean ± SD. RM-ANCOVA, repeated-measures analysis of covariance; PFT, plantar fascia thickness; df, degrees of freedom; *P* FDR, false discovery rate-adjusted *P* value; ηp^2^, partial eta squared.

**Table 3:** Simple-effects analyses and effect sizes for outcomes with significant group × time interactions.

Variable	Comparison	Mean difference	95% CI	Cohen's *d*	*P*	*P*_FDR
VAS	Time 1 Group Diff	−0.63	[−1.03, −0.23]	−0.72	0.002	0.006
	Time 3 Group Diff	−0.93	[−1.43, −0.43]	−0.85	<0.001	0.001
	*Δ*1–0 Diff	−0.5	[−0.89, −0.10]	−0.58	0.015	0.023
	*Δ*3–0 Diff	−0.79	[−1.35, −0.23]	−0.65	0.006	0.011
AOFAS	Time 1 Group Diff	6.36	[3.28, 9.43]	0.95	<0.001	<0.001
	Time 3 Group Diff	7.49	[4.56, 10.41]	1.18	<0.001	<0.001
	*Δ*1–0 Diff	5.32	[1.71, 8.94]	0.68	0.004	0.009
	*Δ*3–0 Diff	6.45	[2.18, 10.72]	0.7	0.004	0.008
YBT	Time 1 Group Diff	0.07	[0.04, 0.10]	0.97	<0.001	<0.001
	Time 3 Group Diff	0.07	[0.05, 0.10]	1.29	<0.001	<0.001
	*Δ*1–0 Diff	0.06	[0.04, 0.09]	1.39	<0.001	<0.001
	*Δ*3–0 Diff	0.07	[0.04, 0.09]	1.1	<0.001	<0.001

Note. Mean differences were calculated as treatment minus control, and change scores were calculated as follow-up minus baseline. *Δ*1–0, change from baseline to 1 month; *Δ*3–0, change from baseline to 3 months; CI, Confidence interval; Cohen's *d*, Standardized mean difference.

### Ankle–hindfoot function (AOFAS scores)

Baseline AOFAS ankle–hindfoot scores were comparable between the two groups (*P* = 0.600). RM-ANCOVA revealed significant main effects of group (*F* = 14.78, *P* < 0.001, *η*p^2^ = 0.064) and time (*F* = 349.39, *P* < 0.001, *η*p^2^ = 0.618), and a significant group × time interaction (*P* < 0.001, *η*p^2^ = 0.063). Simple-effects analyses showed statistically significant between-group differences in AOFAS ankle–hindfoot scores at 1 month, with an adjusted mean difference of 6.36 points (95% CI: 3.28 to 9.43; BH-adjusted *P* < 0.001; Cohen's *d* = 0.95), and at 3 months, with an adjusted mean difference of 7.49 points (95% CI: 4.56 to 10.41; BH-adjusted *P* < 0.001; Cohen's *d* = 1.18) (Tables 2, 3). The clinical importance of these differences remains uncertain.

### Ultrasonographic parameters

Baseline SR and plantar fascia thickness did not differ significantly between the two groups (both *P* > 0.05).

**Figure 5:**
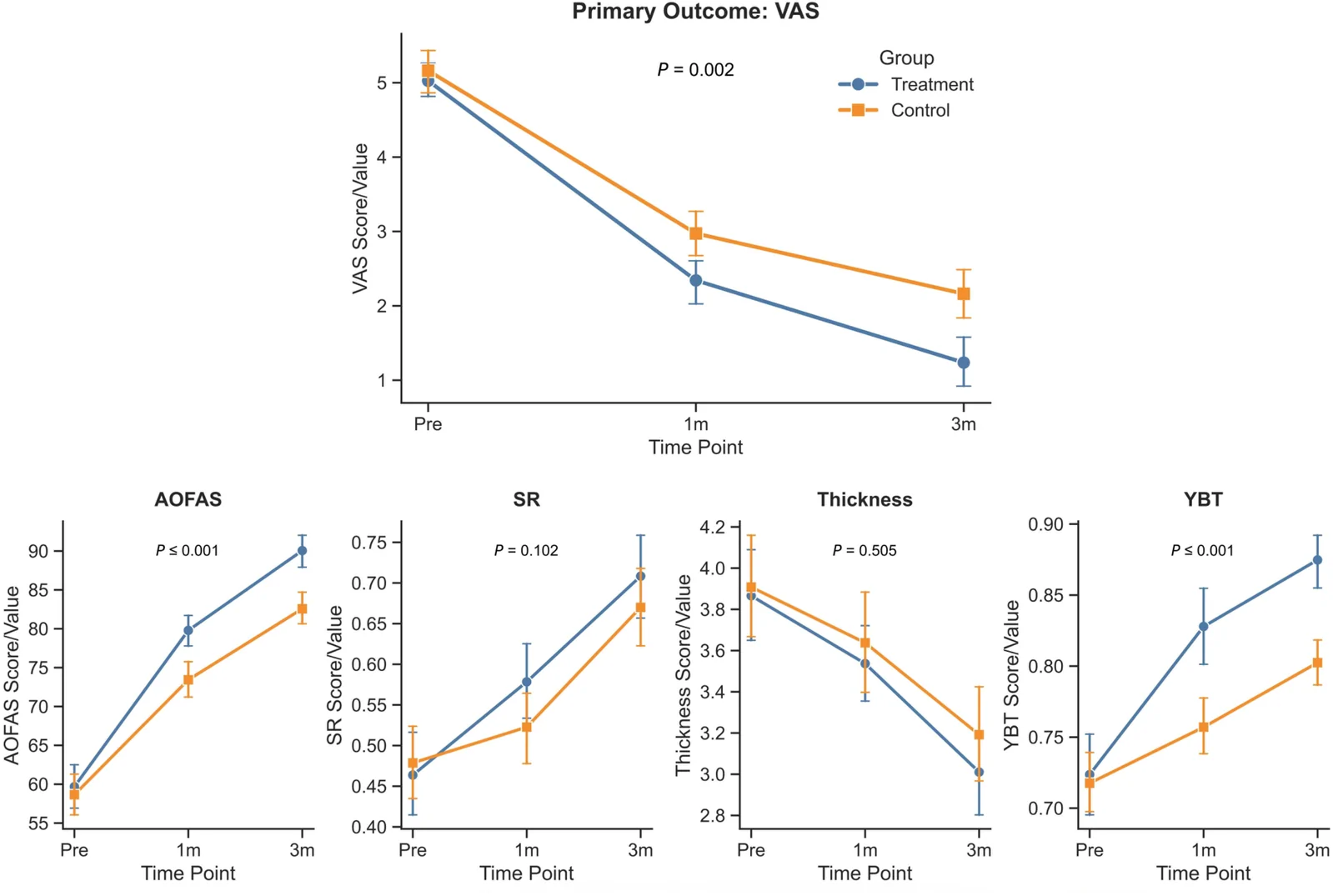
Trajectories of clinical, functional, and ultrasonographic outcomes during follow-up.

For strain ratio (SR), RM-ANCOVA showed a significant main effect of time (*F* = 66.79, *P* < 0.001, *η*p^2^ = 0.236), whereas the main effect of group (*F* = 2.49, *P* = 0.114) and the group × time interaction (*F* = 4.57, *P* = 0.102) were not significant. These findings indicated that SR increased over time irrespective of group allocation, with no evidence of a differential treatment effect between the treatment and control groups (Tables 2, 3; Figure 5).

For plantar fascia thickness, RM-ANCOVA revealed a significant main effect of time (*F* = 72.45, *P* < 0.001, *η*p^2^ = 0.251), whereas the main effect of group (*F* = 0.20, *P* = 0.652) and the group × time interaction (*F* = 1.37, *P* = 0.505) were not significant. These results suggested that plantar fascia thickness decreased over time in the overall sample, but the magnitude of change did not differ significantly between the treatment and control groups (Tables 2, 3; Figure 5).

### Dynamic balance (YBT scores)

Baseline YBT scores were comparable between the two groups (*P* = 0.741). RM-ANCOVA showed significant main effects of time (*F* = 100.07, *P* < 0.001, *η*p^2^ = 0.317) and group (*F* = 19.62, *P* < 0.001, *η*p^2^ = 0.083), and a significant group × time interaction (*F* = 40.18, *P* < 0.001, *η*p^2^ = 0.157). Simple-effects analyses showed that YBT scores improved significantly from baseline in both groups (all *P* < 0.001). Between-group differences were statistically significant at both 1 month and 3 months following treatment. The between-group mean difference was 0.07 at 1 month (95% CI: 0.04 to 0.10; BH-adjusted *P* < 0.001; Cohen's *d* = 0.97) and 0.07 at 3 months (95% CI: 0.05 to 0.10; BH-adjusted *P* < 0.001; Cohen's *d* = 1.29) (Tables 2, 3; Figure 5). The clinical relevance of these YBT differences should be interpreted cautiously.

## Discussion

Plantar fasciitis is a common chronic overuse disorder for which treatment aims include pain relief, functional recovery, and prevention of recurrence. Although ESWT is recognized as an effective treatment for plantar fasciitis (34, 35), conventional protocols mainly target localized plantar-fascia tenderness. This study evaluated a combined ESWT protocol informed by myofascial-chain concepts. The combined protocol was associated with greater short-term improvements in pain, function, and dynamic balance than plantar-focused ESWT alone, consistent with previous evidence supporting ESWT efficacy (36–38). However, the design did not isolate the biological mechanism responsible for these differences. Ultrasound measures, including strain ratio (SR) and plantar fascia thickness, improved over time in both groups but did not differ significantly between groups. Thus, the observed between-group differences were primarily clinical and functional rather than structural on ultrasound. These findings provide preliminary evidence for further evaluation of combined ESWT strategies.

### Clinical significance

The adjusted between-group VAS differences were 0.63 points at 1 month and 0.93 points at 3 months on the 0–10 scale. In plantar heel pain, anchor-based minimal important differences of 0.8 points for average pain and 1.9 points for first-step pain have been reported (39). The 3-month difference slightly exceeded the reported threshold for average pain, whereas the 1-month difference did not; neither difference reached the first-step-pain threshold. Because this trial assessed heel-pain intensity without specifying average pain or first-step pain, these comparisons are approximate. The adjusted AOFAS differences were 6.36 and 7.49 points at 1 and 3 months, respectively. A plantar-fasciitis-specific AOFAS MCID was not identified; estimates from hallux-valgus surgery range from 7.9 to 30.2 points, illustrating that such thresholds are population- and context-dependent (40). Accordingly, statistical significance and standardized effect sizes should not automatically be equated with patient-perceived superiority. Overall, the results indicate a modest additional short-term pain benefit, while the clinical importance of the functional differences remains uncertain.

Studies have described ESWT-related tissue responses including neovascularization, release of growth factors, and collagen remodeling (13–15). Although ESWT is supported by level-B evidence for plantar fasciitis (41), reported success rates vary considerably across studies. Lou et al. (42) reported effectiveness rates ranging from 34% to 88%. Purcell et al. (43) demonstrated that the success rates of extracorporeal shock wave therapy in relieving pain associated with plantar fasciitis also differed widely, from 55% to 88%, with a recurrence rate as high as 20% (16). Myofascial-chain theory has been proposed as one framework for considering continuity within the fascial system. It describes the lower-limb fascia as continuous proximally with pelvic fascia and distally with the plantar fascia through the proposed “Achilles–calcaneal system.” Huijing et al. reported that myofascial expansion between muscles and the epimysial fascia may transmit a proportion of musculoskeletal force (44). This observation provides a possible basis for hypothesizing that lower-limb myofascial abnormalities may influence foot loading; it does not, however, demonstrate that this mechanism causes plantar fasciitis or explains the treatment effect observed here.

Anatomical studies support connective-tissue continuity for some proposed myofascial pathways, but the functional and clinical relevance of these pathways remains incompletely established (45). Evidence for intermuscular force transmission is moderate for some transitions and limited or heterogeneous for others, and direct *in-vivo* evidence remains insufficient to validate a treatment mechanism (46). Therefore, myofascial-chain theory is used here as a hypothesis-generating framework rather than as an established mechanistic explanation.

Previous research has associated gastrocnemius tightness with plantar fasciitis. Sustained abnormal loading may produce local stress concentration, impair tissue repair, and increase symptom recurrence after treatments directed only at the plantar fascia (47, 48). Increased gastrocnemius tension during stance may increase Achilles-tendon tension and, in turn, plantar-fascia loading. Conservative treatments include rest, medication, orthoses, exercise, and fascial stretching; among these, gastrocnemius-stretching exercises may be clinically useful. Siriphorn A et al. (49) reported that gastrocnemius stretching can relieve plantar-fasciitis symptoms. Stretching of the gastrocnemius-AT complex is commonly believed to be a potentially helpful treatment for plantar fasciitis and related foot and ankle pain when gastrocnemius or intrinsic-foot-muscle tightness is present (50–52). The combined intervention may influence plantar-fascia loading, calf-muscle symptoms, nociception, or other proximal–distal interactions. These possibilities are compatible with the clinical findings, but the present trial did not measure mediation or directly test force transmission; therefore, no specific myofascial-chain mechanism can be inferred from the present findings.

Unlike previous studies, this trial combined dynamic-balance and ultrasound outcomes with pain and functional assessments to explore possible effects of the combined therapy. Dynamic balance, assessed using the YBT, showed the largest between-group difference, with a large group × time interaction and the highest Cohen's *d*. This finding indicates greater short-term dynamic-balance performance after combined ESWT; it should not be interpreted as direct evidence of restored neuromuscular activity or validation of a myofascial-chain mechanism. Fascia has been associated with joint stability, movement coordination, proprioception, and nociception (53). Previous studies have proposed a role for myofascial chains in single-leg balance (54). Triceps surae muscles contribute to gait and balance (55). Changes in gastrocnemius symptoms or stiffness may contribute to balance performance (56), but this remains one of several possible explanations for the greater YBT improvement in the treatment group.

A second notable finding was that between-group differences were observed for the clinical and functional outcomes (VAS, AOFAS, and YBT) but not for the ultrasound measures (SR and plantar fascia thickness). Both ultrasound measures changed significantly over time (main effect of time, both *P* < 0.001), consistent with improvement in both groups. These findings are broadly consistent with previous ultrasound-based studies of ESWT for plantar fasciitis (20, 57, 58). The absence of an additional structural effect in the treatment group indicates that ultrasound-detected morphology did not parallel the between-group clinical differences. Although each session delivered 2,000 impulses in both groups, equal total session dosage did not constitute equal plantar-fascia dosage: the treatment group received 1,000 local impulses compared with 2,000 in the control group, representing a 50% lower local mechanical exposure. Because plantar fascia thickness and SR directly interrogate the locally treated tissue, redistribution of half of the impulses to gastrocnemius trigger points may have attenuated the direct mechanical stimulus to the plantar fascia and reduced the between-group contrast in ultrasound-detectable remodeling, even if proximal treatment contributed to improvements in pain, function, and balance through other mechanisms. This interpretation remains a *post hoc* hypothesis rather than evidence of a local dose-response relationship, because the trial was not designed to vary plantar impulse dose independently. Future studies should evaluate the added effect of gastrocnemius treatment while holding the plantar-fascia dose constant. Longer follow-up is also needed to determine whether structural differences emerge and whether the observed clinical differences persist. Clinically, imaging outcomes alone may therefore be insufficient to characterize patient-centered improvement.

This study has several limitations. First, follow-up was limited to 3 months. Consequently, the durability of benefit, recurrence, delayed structural changes, and longer-term safety cannot be determined, and the conclusions should be restricted to short-term comparative effectiveness. Second, bone and plantar-fascia regions of interest were used to calculate the strain ratio, an approach that has not been universally adopted and remains debated (59, 60). In addition, because probe-compression force was not quantitatively controlled during strain elastography, operator-dependent precompression may have introduced variability into SR measurements. Third, myofascial trigger points were identified by palpation, which is susceptible to examiner bias despite efforts to standardize the examination procedure.

## Conclusion

In conclusion, ESWT applied to both gastrocnemius trigger points and plantar tenderness points was associated with greater short-term improvements in pain, ankle–hindfoot function, and dynamic balance than plantar-focused ESWT alone. The clinical importance of some between-group differences remains uncertain. The trial does not establish that these effects were mediated through a myofascial chain, and treatment safety cannot be determined because adverse events were not systematically recorded. The findings therefore support only preliminary short-term comparative effectiveness and require confirmation in trials with strict intention-to-treat analysis, systematic safety monitoring, equal local plantar dosing, and longer follow-up.

## Data Availability

The original contributions presented in the study are included in the article/Supplementary Material, further inquiries can be directed to the corresponding author/s.
